# Chronic Exposure to the *Fusarium* Mycotoxin Deoxynivalenol: Impact on Performance, Immune Organ, and Intestinal Integrity of Slow-Growing Chickens

**DOI:** 10.3390/toxins9100334

**Published:** 2017-10-20

**Authors:** Stephanie S. Chen, Yi-Hung Li, Mei-Fong Lin

**Affiliations:** Department of Animal Science and Technology, National Taiwan University, Taipei 10673, Taiwan; stephanie90122@gmail.com (S.S.C.); r91626006@ntu.edu.tw (Y.-H.L.)

**Keywords:** deoxynivalenol, chicken, spleen, intestine, tight junction, immunohistochemistry, long-term effects

## Abstract

This study investigates the long-term effects of deoxynivalenol (DON) consumption on avian growth performance, on the proliferation, apoptosis, and DNA damage of spleen cells, and on intestinal integrity. Two hundred and eight 5-day-old black-feathered Taiwan country chickens were fed diets containing 0, 2, 5, and 10 mg/kg of DON for 16 weeks. Body weight gain of male birds in the 2 mg/kg group was significantly lower than that in the 5 mg/kg group. At the end of trial, feeding DON-contaminated diets of 5 mg/kg resulted in heavier spleens. Moreover, the increase in DON induced cellular proliferation, apoptosis, and DNA damage signals in the spleen, the exception being female birds fed 10 mg/kg of DON showing reduced proliferation. Expression of claudin-5 was increased in jejunum of female birds fed 2 and 5 mg/kg of DON, whereas decreased expression levels were found in male birds. In conclusion, our results verified that DON may cause a disturbance to the immune system and alter the intestinal barrier in Taiwan country chickens, and may also lead to discrepancies in growth performances in a dose- and sex-dependent manner.

## 1. Introduction

Mycotoxins are fungal metabolites that can contaminate a variety of food and feed ingredients, which may further cause adverse effects on poultry and livestock. Deoxynivalenol (DON) is by far the most prevalent mycotoxin, which mainly occurs in wheat and maize and is produced by the *Fusarium* fungus [1]. According to a survey conducted in the year 2016, DON appeared in 71% of the feedstuffs and feed samples collected worldwide (a total of 11,275 samples tested for DON), with an average concentration of 0.8 mg/kg of the positives, and with maximum values ranging from 0.3 to 49 mg/kg [2]. Therefore, from the perspectives of animal health and performance, more attention should be paid to the fact that animals will frequently face peak and fluctuating mycotoxin levels.

It is acknowledged that the toxicity of DON relies on its epoxide ring positioned at carbon 12–13 [3]. Although the exact mechanisms are not fully understood, it is speculated that the epoxide group is critical for binding to ribosomes, which inhibits protein synthesis, known as the ribotoxic stress effect [4,5]. Further implications include the induction of certain protein kinases and the regulation of downstream genes, thereby affecting the expression of proteins involved in immunomodulation and apoptosis [6,7,8]. It was proposed that DON may also exert its toxicity by inducing oxidative stress [9]. Previous studies suggested that lipid peroxidation and breaking of DNA strands occurred as a consequence of the elevated oxidative stress caused by DON [10]. The toxin targets high proliferating organs and, as a result, alters the function and stability of the digestive and immune systems, which was the main focus of the present study.

Despite the general cytotoxicity of DON, the severity of effects can differ among animal species. In general, the rank order of susceptibility among animals is acknowledged as swine > mice > rats >> poultry ≈ ruminants [11,12]. Therefore, the European Commission established regulatory guideline levels for DON not exceeding 0.9 to 5 mg/kg in feed, depending on the exposed species [13]. While swine are the most sensitive farm animals to DON, poultry seem to have greater tolerance to high doses of DON in terms of performance and productivity [14,15,16]. Previous studies have discussed the underlying mechanisms of this phenomenon. Osselaere et al. [17] found that chickens demonstrated high DON clearance of the body compared to pigs [18]. Further studies confirmed that, following consumption, DON was rapidly metabolized by poultry, which may be attributed to the clearing efficiency of the intestinal mucosa, liver, or possibly kidney [19,20].

Following the ingestion of the contaminated feed, the digestive tract is the first to encounter DON and may be altered by its toxicity. The intestinal villi appeared to be shorter in chickens fed moderate doses (1–5 mg/kg) of DON [21,22]. Whether this phenomenon will subsequently affect nutrient uptake is still inconclusive. Furthermore, DON seemed to impair the barrier function of the intestine through downregulating certain tight junction proteins in pigs [23]. Yet another study found an increasing mRNA expression of claudin-5 (CLDN5) in jejunum of chickens [24]. Since it was hypothesized that DON inhibits protein synthesis by arresting the ribosome [4,5], analyzing the protein expression of CLDN5 on jejunum explants is warranted to acquire relevant translational data.

DON may affect the relative weights of internal organs in broilers such as the heart, liver, and spleen [25,26,27], but the results vary in different breeds, sexes, and feeding conditions. Swelling or atrophy of an organ might reflect inflammation or tissue damage caused by the toxin [28,29]. In addition, elevated activities of serum enzymes, such as aspartate transaminase (AST) and alanine transaminase (ALT), were reported in pigs after chronic exposure to DON, and may be associated with liver damage confirmed by histopathological observations [30]. There was extensive evidence that DON could either be immunosuppressive or immunostimulatory, depending upon the dose and duration of exposure [31]. In vivo and in vitro studies indicated that immune cells are very sensitive to DON [3], as observed in the reduction of lymphocytes or the degraded levels of vaccine antibody titers in poultry [16,27,32]. Oxidative stress may also be induced by DON and resulted in the damaging of DNA in chicken lymphocytes [33,34]. The above studies suggested that DON targets immune cells, which may also involve the alterations of cell proliferation, apoptosis, and DNA damage.

The main interest of the present study was to explore the effects of DON on the avian spleen with long-term feeding in vivo, in which we take the lymphoid organ as a whole, as compared to previous studies mostly focused on the direct effect on certain immune cells conducted in vitro. DON has been reported to induce proinflammatory cytokines, which could directly or indirectly enhance differentiation of IgA-secreting B cells [3,35]. It was acknowledged that the germinal center is the site where antigen-induced B cells mature and differentiate in spleen tissues [36]. The number and sizes of germinal centers could be used to evaluate whether the organism was under stimulation by infectious materials; thus, more information may be revealed by histopathological examination of germinal centers in spleen tissues.

Moreover, for studying the effects of DON on a cellular and protein level, we performed immunohistochemistry (IHC) tests and terminal deoxynucleotidyl transferase dUTP nick end labeling assay (TUNEL assay) on spleen tissues to locate the proliferating, apoptotic, and DNA damaged cells in situ. Proliferating cell nuclear antigen (PCNA) is recognized as a DNA clamp to assist DNA polymerase δ during cell mitosis, and can be used to determine cell proliferation via IHC tests [37]. As for estimating apoptotic cells, TUNEL staining was often used for identifying 3′-hydroxyl ends of fragmented DNA, whose presence was known to be characteristic of cells undergoing apoptotic process [38]. γ-H2AX is considered a sensitive marker for detecting DNA damage in cells [39,40]. If a DNA double-strand break (DSB) occurred, as a part of the histone family, H2AX becomes phosphorylated (also known as γ-H2AX) and migrates adjacent to the site of injury to form a focus, which is visible by immunostaining [39].

Although many studies have evaluated the effects of DON on broilers, there is a lack of information on the slow-growing species. Black-feathered Taiwan country chickens are slow-growing meat-type chickens that produce high-quality meat favored by consumers [41]. They have a relatively long feeding period, with a marketing age of 15–16 weeks and weight of 2.1–2.5 kg [42]. Therefore, they were considered to be a suitable animal model for understanding the long-term effects of DON consumption. To provide more in vivo evidence for the chronic toxicity of DON in chickens, increasing levels of DON were fed to chickens for up until 16 weeks. At several endpoints, we investigated growth performance and DON carryover in livers. We analyzed certain blood biochemical parameters for the estimation of DON effects, which is based on the assumption that liver integrity (AST, ALT), protein utilization (uric acid, UA), and humoral immunity (IgA) may be altered by DON and thus require inspection. Certain phenotypic biomarkers of the spleen, as mentioned above, were assessed via immunohistochemical approaches to understand the impact of DON on cell proliferation, apoptosis, and DNA damage in the avian lymphoid organ. Immunoblotting of intestinal tight junction protein, CLDN5, was performed to evaluate the effect of DON on intestinal barrier. This study aims to provide more insights into in vivo implications of the long-term effects of DON consumption in poultry.

## 2. Results

### 2.1. Dietary Composition, Deoxynivalenol Concentrations, Chicken Performance, and Carryover

Ingredient formulation and nutrient composition of the basal diets for three growing phases are presented in Table 1. Dietary concentrations of DON are given in Table 2. The limit of detection was 0.19 mg/kg. The measured DON levels of 0, 2, 5, and 10 mg/kg were as expected. The detection of other mycotoxins in basal and DON-contaminated diets shows that zearalenone (ZEA) was present as a minor contaminant at a level of 1.9 mg/kg in the 10 mg DON/kg diet, while aflatoxins and fumonisins were of a minuscule amount (Table S1). The initial body weight of chickens among each group was balanced (Table 3). During the experiment, there was no abnormal behavior observed due to consumption of DON, and the death rate was within the range of expectation (mortality of control and each DON treatment was 1–2 birds). The main interest of this study was to investigate the effects of DON; thus, the following results will focus on the findings based on DON treatment, rather than discussing the differences in sexes and/or durations of exposure. In growth performance, there was a significant interaction between DON and sexes (D × S, *p* = 0.002) on body weight gain (BWG), whereas the effect of DON seems to be consistent regardless of the duration of exposure (D × L, *p* = 0.134; D × S × L, *p* = 0.359). Other than this, no alterations of feed intake and feed efficiency were shown due to DON consumption. DON concentrations in liver obtained from 10 mg/kg group at the end of the trial were below the detection limit.

**Figure 1:**
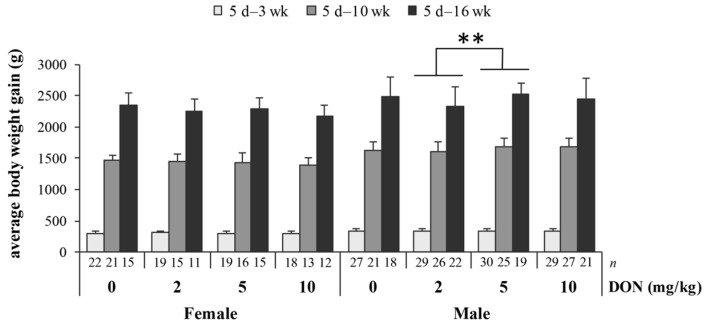
The impact of deoxynivalenol (DON) on growth performance of Taiwan country chickens at different durations of exposure. Bars are presented as mean ± SD; d, wk: days or weeks of age; ** *p* < 0.01.

The distributions of BWG in treatments at varying durations are summarized in Figure 1, classified by sex. Male chickens of 2 and 5 mg/kg groups were significantly different (*p* = 0.004). In addition, BWG of male chickens in the 2 mg/kg group tended to be lower than that in the 10 mg/kg group (*p* = 0.090). For female chickens, no significant differences of control and DON-groups were found throughout the experiment. The fact that DON exerted its effect specifically on BWG of male but not female chickens explains the interaction between DON and sexes.

Because of the difficulty in distinguishing between sexes by appearance up until 10 weeks of age, sampling of birds for further sacrificing turned out to be unequal in sexes. Since there were no significant differences on the parameters of the sampled chickens at three weeks of age, the data of sacrificed birds was not presented. On the other hand, there was insufficient data due to the unequal sampling of sexes at 10 weeks of age; thus, the results were also excluded from this study.

### 2.2. Blood Biochemistry and Relative Organ Weights

**Figure 2:**
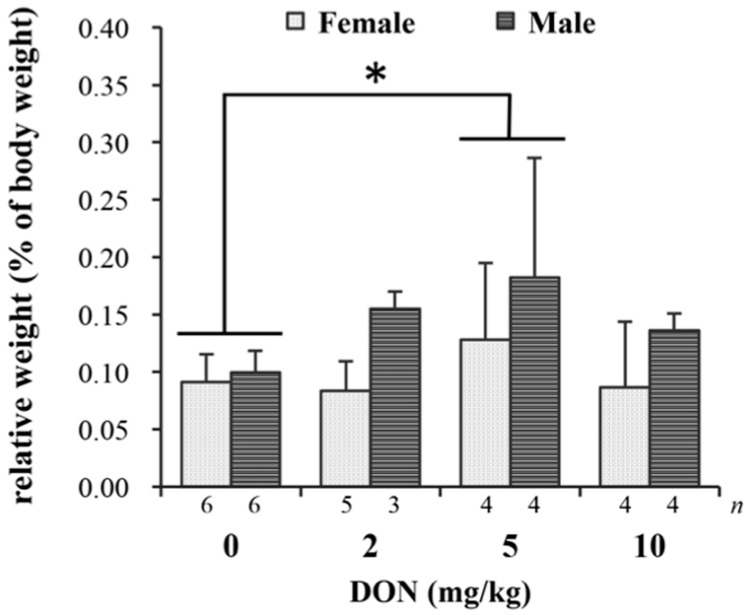
The impact of deoxynivalenol (DON) on relative weight (% body weight) of spleen in Taiwan country chickens at 16 weeks of age. Bars are presented as mean ± SD; * *p* < 0.05.

The detected parameters of blood biochemistry including serum AST, ALT, UA, and IgA levels were not significantly different among treatments (details are presented in Table S2). Relative weights (% of body weight) of liver and heart in chickens fed DON-contaminated diets show no differences (details are given in Table S3). However, splenic relative weight was affected by DON (*p* = 0.038), with 5 mg/kg group being significantly heavier than the control group (Figure 2).

### 2.3. Histopathology, Immunohistochemistry, and TUNEL Assay of the Spleen

**Figure 3:**
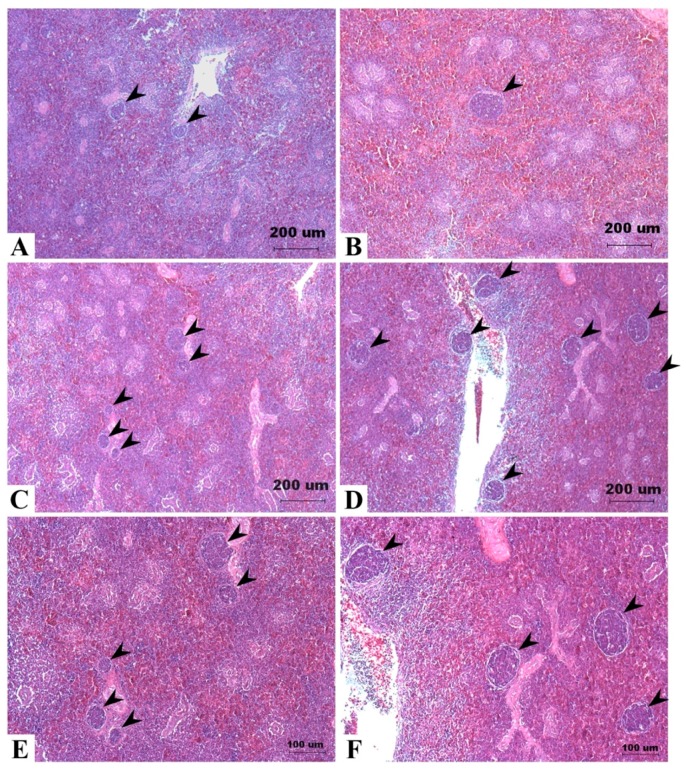
Effects of deoxynivalenol (DON) on spleen histopathology in Taiwan country chickens at 16 weeks of age. Hematoxylin-eosin staining of spleen sections in chickens fed with: (**A**) basal diet; (**B**) 2 mg DON/kg diet; (**C**,**E**) 5 mg DON/kg diet; and (**D**,**F**) 10 mg DON/kg diet. Magnification: (**A**–**D**) ×40; (**E**,**F**) ×100. Germinal centers are indicated by arrowheads.

Histological examination of spleen tissues in Taiwan country chickens fed DON-contaminated diets at 16 weeks of age reveals that the number of germinal centers appeared to be greater in DON groups of 5 and 10 mg/kg (Figure 3).

**Figure 4:**
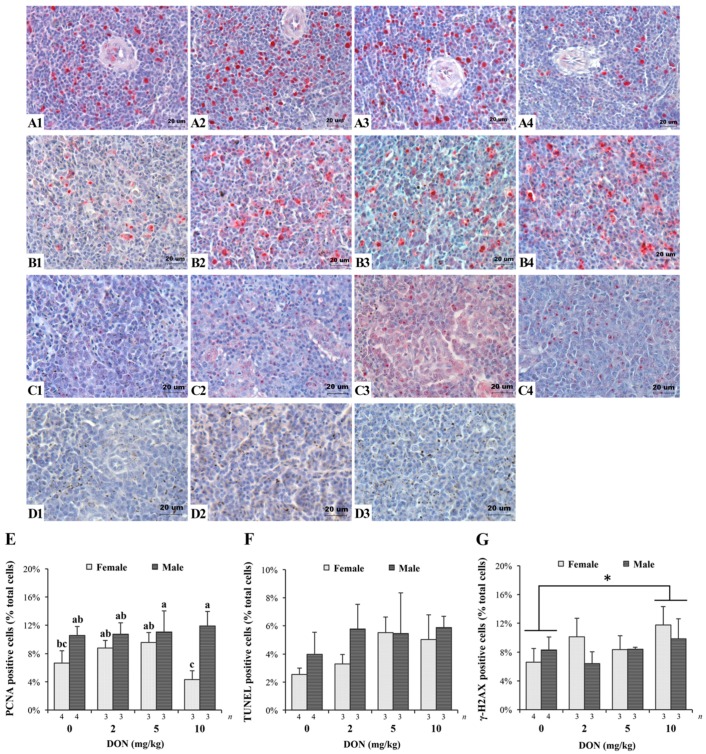
Effects of deoxynivalenol (DON) on spleen proliferation, apoptosis, and DNA damage in Taiwan country chickens at 16 weeks of age: (**A1**–**A4**,**C1**–**C4**) represent spleen sections of immunohistochemical staining of PCNA and γ-H2AX, respectively; (**B1**–**B4**) indicate the TUNEL staining of apoptotic cells; (**D1**–**D3**) represent negative controls of PCNA, TUNEL assay, and γ-H2AX, respectively. Chickens were fed: (**A1**–**C1**) basal diet; (**A2**–**C2**) 2 mg DON/kg diet; (**A3**–**C3**) 5 mg DON/kg diet; and (**A4**–**C4**) 10 mg DON/kg diet; (**E**–**G**) respectively show the percentage of PCNA-, TUNEL-, and γ-H2AX-positive cells in different DON treatments. Magnification: ×400. Positive signals are indicated by magenta color and counterstained by hematoxylin. Bars are presented as mean ± SD; ^a–c^ groups without the same superscript differ significantly at *p* < 0.05; * *p* < 0.05.

The results of IHC and TUNEL assay on spleen tissues of Taiwan country chickens were shown in Figure 4 (see Table S4 for detailed *p*-values). The effects of DON on the proliferation, apoptosis, and DNA damage indexes of the spleen cells were represented by PCNA, TUNEL, and γ-H2AX markers, respectively. A significant interaction exists in measuring PCNA (+) cells (*p* = 0.004; Figure 4E). In general, except for the female chickens in the 10 mg/kg group, PCNA (+) cells gradually increased in DON-consuming group (Figure 4(A1–A3)). Female chickens fed 10 mg/kg of DON had significantly fewer PCNA (+) cells in spleen tissues (Figure 4(A4)). TUNEL assay shows that DON significantly affected the percentage of apoptotic cells (*p* = 0.043), which increased in correlation to the concentration of DON (Figure 4(B1–B4) and F). We also found an increase in γ-H2AX (+) cells of chickens receiving DON-contaminated feed (*p* = 0.048; Figure 4(C1–C4) and G), with 10 mg/kg being significantly higher compared with that of controls. Sections of negative control for these three indicators show no nonspecific binding (Figure 4(D1–D3)).

### 2.4. Intestinal Morphology

**Figure 5:**
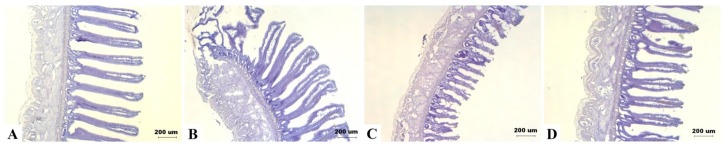
Effects of deoxynivalenol (DON) on the intestinal morphology in Taiwan country chickens at 16 weeks of age. Frozen sections of ileum in chickens fed with: (**A**) basal diet; (**B**) 2 mg DON/kg diet; (**C**) 5 mg DON/kg diet; and (**D**) 10 mg DON/kg diet. Magnification: ×40. Marked difference was noticed in (**C**) as appeared by shortened villi in some male birds.

There was no significant effect of DON diets on villus height of jejunum and ileum (details are given in Table S5). However, there was a tendency of interaction between DON and sexes on ileum height (*p* = 0.079). As shown in intestinal frozen sections, ileum height of some male birds in the 5 mg/kg group appeared to be shorter (Figure 5).

### 2.5. Relative Expression of Tight Junction Protein

**Figure 6:**
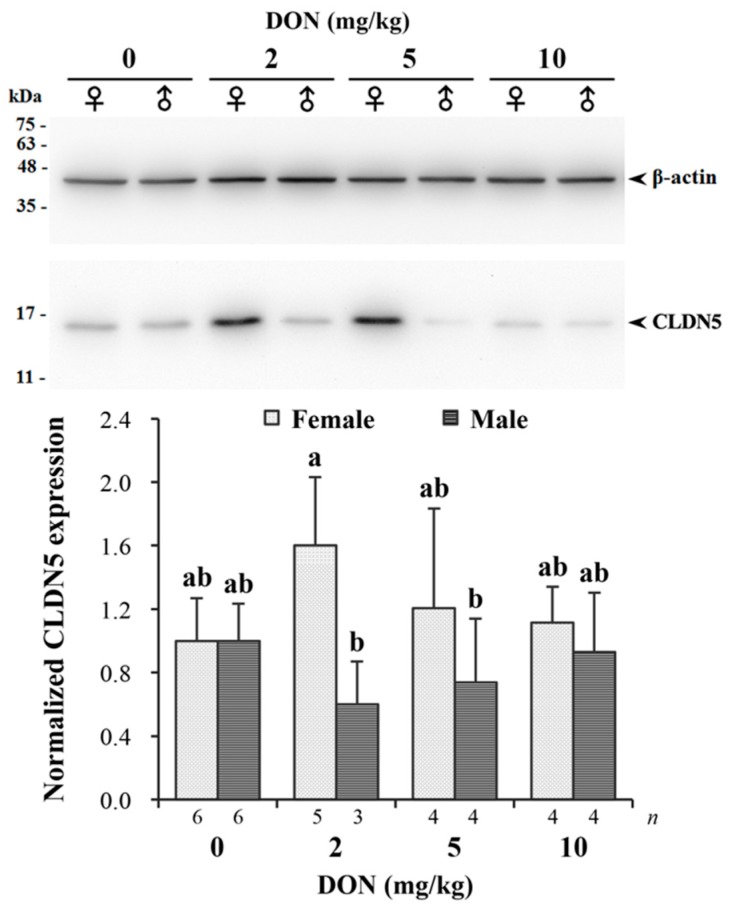
Western blot analysis of claudin-5 (CLDN5) expression in jejunum of Taiwan country chickens fed different levels of deoxynivalenol (DON) contaminated diets at 16 weeks of age. The upper panel illustrates a Western blot representative figure: 10 μg protein from intestinal homogenates were loaded in each lane. The expression of the proteins was estimated by densitometry after normalization with β-actin signal. The lower panel shows normalized CLDN5 expression. Bars are presented as mean ± SD; ^a,b^ groups without the same superscript differ significantly at *p* < 0.05.

Measured relative expression of CLDN5 normalized by β-actin in jejunum is given in Figure 6. The targeting band of CLDN5 with a molecular weight around 17 kDa on Western blotting was comparable to previous studies [43]. There was a significant interaction found in DON and sexes (*p* = 0.043). DON reduced the expression of CLDN5 in male chickens receiving 2 and 5 mg/kg of DON, while female chickens in the same groups had relatively increased expression levels. The difference between males and females was more pronounced in the 2 mg/kg treatment.

## 3. Discussion

Much literature focused on feeding DON to short-living broilers or laying hens only for a brief period of time; however, little information is available on the impact of DON in poultry under chronic exposure. In the present research, raising of Taiwan country chickens reached as long as 16 weeks at marketing age, and it was considered to be a suitable animal model for discovering the chronic effects of DON. The aim of this study was focused on the long-term effects of DON on the immune and intestinal organs of chickens in concentrations that normally occur in nature.

An expanding amount of literature has shown that co-contamination of multiple mycotoxins in cereals is more likely to occur than a single mycotoxicosis, especially as DON and ZEA can both originate from the *Fusarium* species under similar conditions [44,45]. Moreover, a higher toxicological risk due to synergistic and additive interactions between multiple mycotoxins has become highly noticeable, especially in mice and pigs [46,47]. On the contrary, toxicological synergism between DON and ZEA were rarely observed in poultry studies, as it seemed that even high dietary concentrations were not enough to cause detrimental effects on performance or reproduction [32,48,49].

However, Klapáčová et al. [50] previously found that the co-occurrence of DON and ZEA (2.95 and 1.59 mg/kg, respectively) may adversely affect chickens’ protein and mineral metabolism and liver function, as shown by plasma levels of several indicators. The possibility of combined effects of DON-ZEA on poultry at an internal level, rather than external, supports the need for further research using naturally contaminated grains. In the current study, sampling of the 10 mg DON/kg diet shows that the major contaminant was DON and included a lesser amount of ZEA (1.9 mg/kg), which suggests a presence of ZEA at 0.4 mg/kg with respect to 2 mg DON/kg diet when extrapolated proportionally. The dietary ZEA content was far lesser than that of Klapáčová et al. and thus may not be responsible for the effects observed in the present study. Therefore, the significant findings in the following results may be attributable to DON.

Previous studies indicated that the impact of DON on poultry performance was quite trivial compared to that on other animals [14]. In the current study, however, DON levels of 5 and 10 mg/kg seem to slightly stimulate growth of male chickens, whereas 2 mg/kg shows inhibiting effects. Weight differences were more pronounced between 2 and 5 mg/kg groups, suggesting that 10 mg/kg of DON may possess a milder effect despite only showing a tendency. Although few studies have shown growth promotion of DON, Hamilton et al. [51] reported that daily weight gain of laying chicks increased when consuming 4.6 mg/kg of DON for four weeks, which was in accordance with our findings. Moreover, Swamy et al. [48] found that feed intake and weight gain of broilers exposed to 4.7 or 8.2 mg/kg of DON for eight weeks responded in a quadratic fashion. Their findings suggested that 4.7 mg/kg of DON could have nutritive and growth promoting effects in broilers. The quadratic response may be attributed to hormesis, defined as a toxic substance manifesting a stimulatory effect at subinhibitory concentrations; yet, at higher concentrations beyond the nutritive level, toxicological effects become conspicuous [52]. Therefore, the growth promoting response at 5 mg/kg level found by Swamy et al., which is also observed in our study, may be described as hormesis. On the other hand, the less significance of the 10 mg/kg treatment suggests that growth inhibitory effects caused by higher dosage may be counteracting the promoting effect. Such an antagonistic phenomenon was also shown in the weight increment of the spleen in the following discussion.

For male birds, 2 mg/kg of DON seems too low to reach the promoting threshold, and therefore it remained inhibited throughout the experiment. The growth inhibition of DON at low doses was rarely seen in literature conducted on poultry [15]. Nevertheless, the prolonged exposure of DON even at low doses might evoke disturbances in the turnover of proteins, as DON is known to be a potent protein synthesis inhibitor [3,53], thereby hampering growth performance. However, serum UA was not altered due to DON consumption, which may be attributed to the large individual variation. The highly variable results in measuring UA indicate that it might not be suitable for reflecting the impact of DON on protein utilization.

On the other hand, assessed functional indicators of the liver in blood biochemistry (AST, ALT) show no signs of abnormality. Such results imply that DON may not cause damage to the liver in chickens fed DON-contaminated diets up to 10 mg/kg, which was supported by the unchanged weight of the liver. Moreover, DON in liver samples obtained from the highest DON-consuming chickens were below the detection limit, suggesting that after chronic exposure to 10 mg/kg of DON for 16 weeks, the mycotoxin would not accumulate in chicken liver. The low carryover of DON in poultry had been previously tested [54,55,56], showing that DON was rarely retained in blood, organs, or carcasses of poultry. This may be attributed to the high clearing efficiency of the poultry body, as DON was found to be rapidly metabolized to DON-3-sulfate [19,20]. The increased polarity of DON metabolite may facilitate the removal of the toxin via biliary and renal excretion [3,19,57]. The efficacy of DON elimination by the poultry body probably explains their tolerance to DON toxicity, which supports the current results of unaffected performance in female chickens. In addition, feed intake and feed efficiency were not severely impacted, which were in agreement with previous studies [14]; however, whether there were subtle differences of intake between males and females cannot be deduced due to mixed-sex rearing. The cause of the divergent response that appeared in sexes, however, is difficult to interpret at present. The underlying mechanisms of sex differences from physiologic and metabolic aspects require further comparative studies.

At the end of the experiment, the heavier spleen discovered in chickens fed 5 mg/kg of DON might indicate that the immune system was irritated by the toxin, and possibly caused swelling of the immune organ [27]. The increased weight of the spleen might be related to some alterations on a cellular level. Increasing numbers of germinal centers were observed in the spleen of 5 and 10 mg/kg treatments compared with that of 2 mg/kg and control groups. In a previous study, Girish et al. [58] discovered that after feeding turkey poults with 3.9 mg/kg of DON for up to three weeks, the germinal centers increased in spleen sections, accompanied by the reduction of proliferating cells in the intestinal crypt. They speculated that increased penetration of infectious materials due to gut injury caused by DON may further induce immune response, yet splenic weight was not determined in their study. In our study, it is hypothesized that the increasing numbers of germinal centers might be responsible for the heavier spleen. However, there seems to be no effect on serum IgA, which may be due to the large individual differences in measuring IgA. Less uniformity of the observations caused by impure genetic background of Taiwan country chickens was quite inevitable in this study.

The results of IHC tests and TUNEL staining could help us gain insight and understanding of whether DON exerted its effects on spleen cell proliferation, apoptosis, and DNA damage. While proliferating cells were slightly increased in spleen tissues of 2 and 5 mg/kg treatments for female chickens, it occurred at higher doses for male chickens. The enhanced response may also be regarded as hormesis as previously discussed. Furthermore, 10 mg/kg of DON seems to cause adverse impact on female chickens as shown by the abrupt decrease in proliferating cells, which may be related to its protein inhibition effect [53,59]. Ren et al. [34] isolated splenic lymphocyte from chickens and discovered that cultures pretreated with DON had suppressed proliferative activity. Our results provide in vivo evidence and support their findings. Although conflicting results were obtained in previous studies conducted on pigs [60,61,62,63,64], the exposure time length was hypothesized to be accountable for the inconsistent findings. Pinton et al. [65] observed an antigenic stimulation of lymphocyte proliferation by ovalbumin after feeding pigs three weeks of DON-contaminated feed, and further found suppressed responses through 5–7 weeks of exposure. We can speculate that the toxicological effects of DON may accumulate through prolonged exposure. The stimulation of proliferating cells by DON in their study agrees with our findings, while suppressed response induced by cumulated toxin may as well explain the decreased proliferation in chickens fed the highest DON level.

As for detecting apoptotic cells, we found that the higher the DON dose given, the more apoptotic cells existed in spleen tissues. It was previously reported that DON-treated lymphocytes obtained from chicken spleen showed upregulated expressions of apoptosis-related genes under in vitro survey [34]. The cytotoxicity of DON had been proven in extensive work, and apoptotic genes (Bax, Bak-1, p53, Caspase-3) regulated by mitogen-activated protein kinases (MAPKs) may be deeply involved in the consequences [3]. Our results once again supply in vivo evidence and confirmed previous research.

Other than apoptosis, we also found greater γ-H2AX signals in spleen tissues of chickens consuming DON-contaminated diets. Typically, the number of foci formed in cells agrees with the yield of every DSB [66]. Marked numbers of foci were easily distinguished in cell nucleus of the 10 mg/kg treatment, implying greater DNA damage. DNA containing nitrogen base with double bands are easy targets for reactive oxygen species (ROS), thus DNA damage may be related to the elevated oxidative stress caused by DON [9]. Ren et al. [34] showed that, by culturing DON with chicken splenic lymphocytes in vitro, the toxin significantly induced ROS accumulation in cells, and simultaneously caused DNA fragmentation. If DSBs become more severe, it may further lead to apoptosis; therefore, the patterns of spleen sections retrieved by TUNEL assay had close relations with γ-H2AX immunostaining.

Taken together, we can speculate that DON could stimulate proliferation, and at the same time, trigger apoptosis of the immune organ in chickens. Moderate–low levels of DON may promote cell proliferation, whereas, at higher doses, it caused greater DNA damage and apoptosis in chickens, as well as suppressed proliferation specifically in female birds. Our findings were consistent with related studies describing the biphasic impact of DON, which suggested both immunostimulative and immunosuppression effects [3,31,67,68]. The stimulation of proliferation under moderate–low doses of DON may be correlated to the weight increment of spleen in the 5 mg/kg group, while the unaffected spleen weight at high DON dosage implies that it might be undergoing a transitional stage, supported by the cellular evidence that negative impact occurred at higher levels. In addition, Swamy et al. [69] pointed out that time of toxin exposure may be an important factor in organ weights, since the organ initially swells with short-term exposure, followed by shrinkage with long-term exposure. Predictably, spleen of chickens consuming higher DON doses may progress toward shrinkage, according to the signs as shown above. It should also be mentioned that one of the limitations of this study is the difficulty in identifying different lymphocytes of spleen specimens. Whether DON specifically alters certain cell types cannot be determined from the manifested results, and should be clarified in further research.

Regarding the effect of DON on intestinal morphology, while duodenum samples were distorted due to mishandling and thus cannot be compared, it seems that villus height of jejunum and ileum explants were not significantly different among treatments. However, we discovered a tendency of interaction between DON and sexes in ileum during 16 weeks of age. Marked difference was noticed in some male chickens of the 5 mg/kg treatment having obviously shorter villi, which merely reached half the height of controls in comparison. Such observation suggests that there may be some susceptible individuals affected by its toxicity.

Our results differ from that of previous studies, which mostly observed that DON altered the morphology of the upper small intestine; specifically, the villi of duodenum and jejunum appeared to be shorter [15]. Nevertheless, a feeding trial conducted by Yunus et al. [70] revealed that the irritant effects of DON progressively changed the small intestine of broilers from the proximal to distal tract through four weeks of exposure. In the present study, the decreased villi height of ileum in male chickens may be developed through exposure time as long as 16 weeks. In comparing villi height of jejunum, marked individual variation may be accountable for the insignificant results, yet we cannot exclude the possibility that different intestinal segments may differ in sensitivity to DON toxicity.

Some authors speculated that the morphological and functional adaptations of the chicken intestine could be associated with the decreased systemic DON absorption after chronic exposure by reducing the absorptive surface [57]. Moreover, Yunus et al. [70] hypothesized that the lack of adverse effects on nutrient digestibility over the entire tract might be due to the increased length of the small intestine after chronic exposure. This phenomenon may also in turn be conducive to microbial detoxification of DON in the gut, supported by the proof that higher deepoxidation activity in intestinal bacteria was retrieved from DON-feeding hens [71]. These compensatory adaptations observed in chickens might in part explain its high tolerance to DON.

The intestinal epithelium plays an important role in selecting external molecules and regulating the absorption of nutrients, while acting as an effective barrier against toxins, antigens, and enteric pathogens [72]. The maintenance of intestinal integrity relies on the formation of complex protein networks in adjacent epithelial cells to seal the intercellular space, one of which includes the tight junction proteins [73]. Pinton et al. [23] conducted a thorough research applying in vitro, ex vivo, and in vivo approaches of pig models to assess the effects of DON on the intestinal epithelium. Their results revealed that DON reduced the expression of tight junction proteins (especially claudins) and intensified bacterial translocation across epithelial monolayers, which implied that DON increased intestinal permeability.

However, to our knowledge, studies on the impact of DON on intestinal barrier in poultry models are limited. In the present study, the increasing CLDN5 protein expression of female chickens in 2 and 5 mg/kg groups are consistent with the former work by Osselaere et al. [24], who observed an upregulation of CLDN5 mRNA in broilers fed DON diets. The exact reasons for CLDN5 upregulation by DON cannot be ascertained from the data presented; however, it could be considered as a protective mechanism. It was reported that once the barrier function of the intestine was seriously impaired by chronic exposure, increasing bioavailability of DON was observed in pigs, which may further lead to greater susceptibility to infectious materials [18]. On the other hand, chickens seemed to gain greater tolerability to DON after chronic exposure, as shown by lower systemic concentrations of the toxin [70,74]. Therefore, we consider that the increased expression of CLDN5 in chicken intestine may be regarded as an adaptive response to maintain intestinal barrier against further DON damage, which was developed through long-term exposure. This finding is paralleled by the lack of DON effect on the performance of female birds in the 2 mg/kg treatment, which, in part, could explain the divergent response of sexes in the same group. The decreased expression levels of male chickens in 2 and 5 mg/kg groups may result from the inhibition effect of DON on protein synthesis, as approved by many others [75]. Increased intestinal permeability of male chickens in the 2 mg/kg group may further result in greater sensitivity to DON, thus simultaneously causing poor performance. In contrast, for male birds fed 5 mg/kg of DON, reduced absorptive surface of shorter villi may be antagonistic to the altered permeability of tight junctions, thereby overcoming the impact of DON on growth performance.

## 4. Conclusions

From our findings, DON exhibited its biphasic effects in a dose- and sex-dependent manner. For male chickens, growth promotion was noticed at certain threshold levels (5 mg/kg), whereas prolonged exposure to DON at the level of 2 mg/kg resulted in growth inhibition. Moderate–low levels of DON (2 and 5 mg/kg) may alter the barrier function of the intestine in male chickens. Such impact might increase animal susceptibility and be linked to the hindered performance (2 mg/kg). Gut injuries may indirectly lead to irritation of the immune system, as observed by splenic weight increment and germinal center formation (5 mg/kg). Chronic exposure of moderate–high DON doses shows negative impact to the immune organ, leading to greater apoptosis and DNA damage. In addition, female chickens seem to be more sensitive due to inhibited splenic proliferation when fed 10 mg/kg of DON, but there was a lack of DON effect on growth performance. However, it is important to reiterate the fact that zootechnical traits might not precisely reflect the severity of chronic high-DON exposure during the transitional stage. Considering the possible synergistic effect of toxins and pathogens, further investigations are needed to confirm whether such DON impact to the immune and digestive systems would cause poor performance when faced with immunological and environmental stress.

## 5. Materials and Methods

The care and use of experimental animals for this study followed the regulations of Institutional Animal Care and Use Committee (IACUC), approved by the Research Ethics Office of National Taiwan University. Ethical approval code: NTU-101-EL-25; valid from 1 January 2012 for 2 years.

### 5.1. Experimental Design, Birds, and Diets

Two hundred and eight 1-day-old black-feathered Taiwan country chickens were obtained from a commercial hatchery in Changhua, Taiwan, and reared in floor pens of the poultry house located at National Taiwan University. After five days of acclimatizing, chickens were weighed at five days of age and assigned to four treatments based on similar group weight. Thirteen chickens per pen were raised together in mixed-sex, and fed diets containing 0, 2, 5, or 10 mg/kg of DON (four pens per treatment). Feeding and housing managements followed the instructions of the farming industry.

The feeding trial was divided into three exposure time lengths, beginning from five days of age, lasting to 3, 10, and 16 weeks of age. Basal diets for three phases (starter, grower, and finisher) were formulated according to Wang et al. [76] and prepared for all treatments in the same process. The mycotoxin was produced by inoculating brown rice with *F. graminearum*. After 35 days of solid-state fermentation, the product was the contaminated rice with highly concentrated DON at approximately 900 mg/kg. Contaminated diets were prepared by replacing dietary rice with contaminated rice at different levels (see details in Table 1). To analyze the concentration of DON, diets were extracted by polyethylene glycol (PEG) dissolved in water (1/20, *w/v*), and cleaned up with immunoaffinity columns (DONtest HPLC Columns; VICAM, Milford, MA, USA) based on the manufacturer’s instructions, followed by HPLC. Representative feed samples collected from basal and 10 mg DON/kg diets were outsourced for other mycotoxins analysis, such as aflatoxins (B1, B2, G1, G2), zearalenone, and fumonisins (B1 and B2), by Animal Technology Institute Taiwan, Miaoli, Taiwan. Dry matter, ether extract, crude fiber, and crude protein content in feed were determined by approximate analysis according to the standard procedures of Association of Official Analytical Chemists [77].

### 5.2. Sampling and Tissue Collection

At the end of each duration (3, 10, and 16 weeks of age), chickens were weighed individually. Feed intake of each pen was recorded (four pens per treatment) and expressed as average consumption per bird. Feed efficiency was defined as summation of BWG of total chickens divided by total feed intake per pen. For further sampling, we randomly selected two birds per pen (eight chickens per treatment) at the end of each duration; three birds per pen were chosen for the control group at 16 weeks of age.

Blood samples were collected for the analysis of blood biochemistry, such as serum AST, ALT, and UA, which were measured spectrophotometrically using commercial strips with an autoanalyzer (Arkray, Kyoto, Japan). The concentration of IgA in serum was determined by a chicken IgA ELISA quantitation set (Bethyl Laboratories Inc., Montgomery, TX, USA.). Chickens were sacrificed via electrocution, followed by weighing of organs including the heart, liver, and spleen. The middle segments of jejunum and ileum of the intestine were taken at about 3 cm in length, immersed in fixative and transferred to 30% sucrose for long-term storage before sectioning. Tissue samples of jejunum were collected and immediately frozen in liquid nitrogen, and stored at −80 °C before protein analysis. At the end of 16 weeks, spleen samples were removed wholly and immersed in fixative for sectioning. To evaluate the carryover of DON in chicken liver at the end of trial, livers of sampled chickens from the 10 mg/kg group were mixed together for each pen into four portions. Each portion was homogenized with an equivalent weight of water, and added with PEG in adequate amounts (as described above; the water content of liver was estimated to be 80%). The following procedures were the same as analyzing DON concentrations in diets. Chemicals used in the experiment were of analytical grade.

### 5.3. Spleen Sectioning and Staining

After fixation, spleen samples were dehydrated, cleared, paraffin embedded, and sectioned at 4-μm thickness according to the routine method established by Lillie [78] with several modifications. Samples placed on glass slides were stained by hematoxylin and eosin (H&E) for further histopathological examination under light microscopy.

#### 5.3.1. Immunohistochemical Evaluation

The methods from Shi et al. [79] and Ezaki [80] were adopted for antigen retrieval with minor modifications. After deparaffinization, antigen retrieval was carried out under 80 °C of sodium citrate buffer (100 mM sodium citrate, 0.05% Tween-20, pH 6.0) for 50 min. The pretreatment was followed by cooling and rinsing in water and phosphate buffered saline (PBS) prior to IHC tests. IHC assays of detecting PCNA and γ-H2AX signals on the spleen tissue were performed according to the manufacturer’s instructions (Vectastain ABC-AP kit; Vector Laboratories, Burlingame, CA, USA). Sections were incubated with primary antibodies, a mouse monoclonal anti-PCNA (sc-56; Santa Cruz Biotechnology, Santa Cruz, CA, USA) or a rabbit polyclonal anti-γ-H2AX (ab11174; Abcam Inc., Cambridge, MA, USA) at dilutions of 1:500 and 1:1000, respectively. As a negative antigen control, another specimen was incubated with mouse or rabbit nonspecific IgG (Vector Laboratories). Following the incubation of secondary antibodies (Vector Laboratories) and enzyme linking, protein targets were visualized in magenta color by reacting with the alkaline phosphatase substrate (Vector Laboratories). Slides were then counterstained with hematoxylin and mounted for further inspection.

#### 5.3.2. Detection of Apoptosis

To detect apoptotic cells of spleen specimens, TUNEL assay was performed based on previous studies [38,81,82] with modified procedures. In brief, tissue sections were pretreated similarly as IHC, but the retrieval process lasted 30 min and was proceeded by incubation with proteinase K solution (20 μg/mL proteinase K (Sigma-Aldrich, St. Louis, MO, USA) dissolved in 50 mM Tis-HCl, 1 mM ethylenediaminetetraacetic acid (EDTA), pH 8.0) in a humidified chamber at 37 °C for 10 min. After several washes of PBS and blocking of nonspecific binding sites (Avidin/Biotin Blocking kit; Vector Laboratories), 3% bovine serum albumin (BSA) in PBS was applied 30 min for thorough blocking. The specimens were then incubated with reaction buffer (25 mM Tris-HCl, 0.2 M sodium cacodylate, 0.25 mg/mL BSA, 1 mM cobalt chloride) for 20 min. Afterwards, specimens were covered by DNA labeling solution (0.38 U/μL terminal transferase (TdT enzyme; Sigma-Aldrich), 0.32 mM brominated dUTP (BrdUTP; Sigma-Aldrich) dissolved in reaction buffer) and kept in chamber for 2 h. A specimen treated with DNA labeling solution without the TdT enzyme represented the negative control. Slides were rinsed thoroughly, and applied again with blocking buffer for 30 min. Specimens were reacted 1 h with the biotinylated mouse anti-BrdU monoclonal antibody (B35138; Molecular Probes Inc., Eugene, OR, USA), diluted 1:50. The following procedures were basically the same as IHC, in which conjugates and enzyme substrate were applied to provide the magenta color.

#### 5.3.3. Image Analysis

Spleen sections stained by IHC and TUNEL assay were analyzed in two fields per slide by manual counting under ×400 magnification, aided by ImageJ software (National Institutes of Health, Bethesda, MD, USA). Target signals for PCNA (proliferating) and TUNEL (apoptotic) labeled cells were shown by nuclei appearing magenta color, while γ-H2AX signals (locating DNA strand breaks) were presented as foci formed in nuclei. Both positive and negative (bluish-purple color) cells were quantified and results were expressed as percentage of positive cells.

### 5.4. Intestinal Morphology

For the handling and sectioning of intestinal specimens, we referred to the method described by Ozden et al. [43] with some modifications. In brief, stored samples were blotted, trimmed, embedded within OCT compound (Tissue-Tek O.C.T. compound; Sakura Finetek, Torrance, CA, USA), and frozen in liquid nitrogen. Tissue blocks were frozen-sectioned using a cryostat microtome (Leica, Heidelberg, Germany) at 8-μm thickness and placed on glass slides. Sections were stained by hematoxylin and observed by light microscopy under ×40 magnification. Photos were taken and measured by Image-Pro AMS 6.0 software (Media Cybernetics Inc., Silver Spring, MD, USA). A total of twenty intact villi of jejunum or ilium were measured in height for an individual chicken.

### 5.5. Tissue Protein Extraction, Separation, and Western Blotting

The expression of CLDN5 in jejunum was analyzed according to González-Mariscal et al. [83] with some adjustments. Total protein of frozen sample was extracted by lysis buffer (RIPA Lysis Buffer; Merck Millipore, Temecula, CA, USA) containing protease inhibitors (1 mM PMSF, 1 mM Na_3_VO_4_, 1 mM NaF, 25 mM β-glycerophosphate and 0.5% protease inhibitor cocktail (Merck Millipore)). Lysate was centrifuged and the supernatant was homogenized through a 26 G needle. Samples were then sonicated with intermittent cycles and centrifuged again to obtain the protein extract. Proteins were quantified by Bradford assay [84] using commercial reagents (Bio-Rad, Hercules, CA, USA). Extracts were mixed with sample buffer (50 mM Tris-HCl pH 6.8, 10% glycerol, 5% β-mercaptoethanol, 2% SDS, 0.1% bromophenol blue) to a final protein concentration of 1 μg/μL, and heated at 95–98 °C for 10 min.

Protein separation and immunoblotting were carried out by employing the frequently practiced Bio-Rad systems (Mini-PROTEAN Tetra Cell and Mini Trans-Blot Electrophoretic Transfer Cell; Bio-Rad), following the manufacturer’s instructions. In brief, a total of 10 μg protein from jejunum tissue was separated by sodium dodecyl sulphate-polyacrylamide gel electrophoresis (SDS-PAGE) with 15% running gel, and transferred onto polyvinylidene difluoride membranes (Merck Millipore). Membranes were cut into two-halves with the target protein on one side and β-actin, used as an internal control, on the other. Membranes were then blocked with 5% skimmed milk in Tris-buffered saline with Tween-20 (TBST) for 1 h, followed by incubation (4 °C, overnight) with primary antibodies, a mouse monoclonal anti-β-actin (MA5-15739; Thermo Fisher Scientific, Rockford, IL, USA) or rabbit polyclonal anti-CLDN5 (SAB2100442; Sigma-Aldrich), diluted 1:1000 in blocking buffer. After several washes, they were incubated with horseradish peroxidase (HRP) conjugated secondary antibodies (Thermo Fisher Scientific) at dilutions of 1:5000 for 1 h. Bindings of antibodies were detected with the chemiluminescent HRP substrate (Merck Millipore) after placing the membranes against UVP imaging systems (VisionWorks LS Image Acquisition and Analysis Software; UVP, Upland, CA, USA). The intensities of developed bands were estimated by ImageJ software (National Institutes of Health). The expression of CLDN5 was normalized by β-actin and presented as relative protein expression. Values were calibrated by the mean of the control group.

### 5.6. Statistical Analysis

Data analysis was conducted by using the SAS statistical software package, version 9.3 (SAS Institute, Cary, NC, USA). The main effects of DON (0, 2, 5, and 10 mg/kg), sex (male and female), exposure time length (duration: 5 days–3 weeks, 5 days–10 weeks, and 5 days–16 weeks of age), and their interactions were determined by adopting the general linear model (GLM) computing procedure for ANOVA, followed by the Tukey–Kramer’s multiple comparisons test among least square means (LSMEANS) for unbalanced samples. Due to the excluded data of sacrificed birds at 3 and 10 weeks of age, statistical analysis of data from sacrificed birds at 16 weeks of age was done by using only DON and sex as the main effects. A statistical level of *p* < 0.05 was used as the criterion for significance, while *p* < 0.1 defined tendency.

In consideration of previous studies that indicated the strong correlation between chick weight and both growing performance and the development of internal organs [85,86,87], the analysis of covariance (ANCOVA) was employed for analyzing the BW, BWG, and organ weights. The above parameters were adjusted for initial body weight at five days of age as a covariate by covariance analysis, if the covariate showed statistical significance of *p* < 0.2.

## Figures and Tables

**Table 1:** The composition of basal diets.

Ingredient (g/kg as Fed Basis)	Diet		
	I	II	III
Corn	510	570	600
Soybean meal	360	320	310
Fish meal	25	15	-
Soybean oil	55	45	39
Brown rice ^1^	15	15	15
Monocalcium phosphate	15	11	11
Limestone	11	15	15
Iodized salt	3	4	4
DL-Methionine	2	2	2
Vitamin Premix ^2^	1	1	1
Mineral Premix ^3^	1	1	1
Choline chloride, 50%	1	1	1
Total	1000	1000	1000
Calculated value			
Metabolizable Energy (kcal/kg)	3159	3150	3146
Crude Protein (g/kg)	220.1	200.1	186.9
Analyzed value			
Dry Matter (g/kg)	887.1	884.7	886.1
Crude Protein (g/kg)	224.6	207.7	191.2
Ether Extract (g/kg of DM)	90.6	81.2	70.1
Crude Fiber (g/kg of DM)	34.9	30.2	33.1

DM: dry matter; I–III: growing phase of 5–21, 22–70, and 71–112 days of age. ^1^ The incorporation of deoxynivalenol (DON)-contaminated rice (900 mg/kg) into DON diets of treatments 2, 5, and 10 mg/kg were stepwise at proportions of 15%, 37%, and 74% of total dietary rice; ^2^ Supplied per kg diet: Vit A 12,000 IU; Vit D_3_ 3000 IU; Vit E 38 IU; Vit K 3 mg; Thiamin 2.2 mg; Riboflavin 7.2 mg; Pyridoxine 4.5 mg; Vit B_12_ 0.018 mg; Niacin 50.0 mg; Ca-Pantothenate 14.0 mg; Folic acid 1 mg; Biotin 0.22 mg; ^3^ Supplied per kg diet: Mn 65.9 mg; Zn 58.9 mg; Cu 16.3 mg; Fe 59.3 mg; Se 0.22 mg; Co 0.27 mg.

**Table 2:** The analyzed concentrations of deoxynivalenol (DON) in diets (mg/kg).

Treatment	Diet		
	I	II	III
Basal diet	ND	ND	ND
2 mg/kg	2.39	2.21	2.07
5 mg/kg	5.17	5.27	5.31
10 mg/kg	10.88	10.01	10.92

ND: not detectable; I–III: growing phase of 5–21, 22–70, and 71–112 days of age.

**Table 3:** The impact of deoxynivalenol (DON) on growth performance of Taiwan country chickens.

Age	Treatment (DON, mg/kg)								*p*-Values ^1^						
	0		2		5		10								
	♀	♂	♀	♂	♀	♂	♀	♂	D	S	L	D × S	D × L	S × L	D × S × L
Body weight (g)															
Initial (5 d)	49.3 ± 5.2 (*n* = 24)	49.4 ± 4.8 (*n* = 28)	48.8 ± 5.0 (*n* = 20)	49.5 ± 4.8 (*n* = 30)	50.9 ± 3.9 (*n* = 20)	48.1 ± 5.0 (*n* = 32)	48.9 ± 5.2 (*n* = 19)	49.4 ± 4.3 (*n* = 31)	0.999	0.629	-	0.247	-	-	-
3 wk	348 ± 41 (*n* = 22)	383 ± 33 (*n* = 27)	356 ± 37 (*n* = 19)	387 ± 43 (*n* = 29)	354 ± 32 (*n* = 18)	384 ± 40 (*n* = 30)	350 ± 31 (*n* = 17)	388 ± 38 (*n* = 29)	0.120	<0.0001	<0.0001	0.002	0.135	<0.0001	0.350
10 wk	1509 ± 96 (*n* = 21)	1681 ± 130 (*n* = 21)	1505 ± 112 (*n* = 15)	1654 ± 162 (*n* = 26)	1480 ± 151 (*n* = 16)	1724 ± 148 (*n* = 25)	1435 ± 114 (*n* = 13)	1739 ± 141 (*n* = 27)							
16 wk	2399 ± 203 (*n* = 15)	2526 ± 311 (*n* = 18)	2301 ± 183 (*n* = 11)	2386 ± 302 (*n* = 22)	2332 ± 178 (*n* = 15)	2569 ± 181 (*n* = 19)	2218 ± 174 (*n* = 12)	2498 ± 329 (*n* = 21)							
Feed intake (g/bird)															
5 d–3 wk	519 ± 18 (*n* = 4)		526 ± 19 (*n* = 4)		520 ± 18 (*n* = 4)		538 ± 27 (*n* = 4)		0.288	-	<0.0001	-	0.405	-	-
5 d–10 wk	3727 ± 179 (*n* = 4)		3676 ± 198 (*n* = 4)		3719 ± 191 (*n* = 4)		3727 ± 61 (*n* = 4)								
5 d–16 wk	8507 ± 474 (*n* = 4)		7992 ± 406 (*n* = 4)		8391 ± 356 (*n* = 4)		8225 ± 281 (*n* = 4)								
Feed efficiency															
5 d–3 wk	0.61 ± 0.03 (*n* = 4)		0.61 ± 0.02 (*n* = 4)		0.60 ± 0.02 (*n* = 4)		0.59 ± 0.02 (*n* = 4)		0.871	-	<0.0001	-	0.654	-	-
5 d–10 wk	0.42 ± 0.00 (*n* = 4)		0.42 ± 0.02 (*n* = 4)		0.43 ± 0.01 (*n* = 4)		0.43 ± 0.01 (*n* = 4)								
5 d–16 wk	0.28 ± 0.01 (*n* = 4)		0.29 ± 0.02 (*n* = 4)		0.29 ± 0.01 (*n* = 4)		0.29 ± 0.01 (*n* = 4)								

Values are presented as mean ± SD; d, wk: days or weeks of age. ^1^ The calculated *p*-values using DON (D), sex (S), and exposure time length (L) as the main effects in factorial model.

## Supplementary Materials

The following are available online at www.mdpi.com/2072-6651/9/10/334/s1, Table S1: The analyzed concentrations of several mycotoxins in diets; Table S2: The impact of deoxynivalenol (DON) on blood biochemistry in serum of Taiwan country chickens at 16 weeks of age; Table S3: The impact of deoxynivalenol (DON) on relative weights (% of body weight, BW) of liver, heart, and spleen in Taiwan country chickens at 16 weeks of age; Table S4: Detection of cell proliferation marker PCNA, apoptosis signals by TUNEL assay, and DNA damage related marker γ-H2AX in spleen sections of Taiwan country chickens exposed to deoxynivalenol (DON) at 16 weeks of age; Table S5: The impact of deoxynivalenol (DON) on villus morphology of the small intestine in Taiwan country chickens at 16 weeks of age.
