# Supplementary material for: Chronic Exposure to the Fusarium Mycotoxin Deoxynivalenol: Impact on Performance, Immune Organ, and Intestinal Integrity of Slow-Growing Chickens
Source: Toxins (Basel). 2017 Oct 20;9(10):334. doi: 10.3390/toxins9100334 (PMC5666380; doi:10.3390/toxins9100334)
Supplement: Supplementary file 1 [file toxins-09-00334-s001.pdf]

Supplementary Materials: Chronic exposure to the *Fusarium* mycotoxin deoxynivalenol: impact on performance, immune organ, and intestinal integrity of slow-growing chickens

Stephanie S. Chen, Yi-Hung Li and Mei-Fong Lin

Table S1. The analyzed concentrations of several mycotoxins in diets.

Mycotoxins	Basal diet	10 mg DON/kg diet
Aflatoxin B1 (μg/kg)	< 0.30	< 0.30
Aflatoxin B2 (μg/kg)	ND	ND
Aflatoxin G1 (μg/kg)	ND	ND
Aflatoxin G2 (μg/kg)	ND	ND
Zearalenone (μg/kg)	ND	1989.80
Fumonisin B1 (mg/kg)	0.07	0.04
Fumonisin B2 (mg/kg)	0.03	0.03

ND: not detectable.

Table S2. The impact of deoxynivalenol (DON) on blood biochemistry in serum of Taiwan country chickens at 16 weeks of age.

Parameters	Treatment (DON, mg/kg)								*p*-Values [1]		
	0		2		5		10				
	♀	♂	♀	♂	♀	♂	♀	♂	D	S	D × S
AST (IU/L)	207 ± 75 (*n* = 6)	215 ± 42 (*n* = 6)	137 ± 9 (*n* = 5)	196 ± 27 (*n* = 3)	163 ± 28 (*n* = 4)	178 ± 28 (*n* = 4)	161 ± 33 (*n* = 4)	199 ± 11 (*n* = 4)	0.059	0.060	0.566
ALT (IU/L)	12.2 ± 8.3 (*n* = 6)	14.4 ± 6.8 (*n* = 6)	13.0 ± 6.3 (*n* = 5)	14.5 ± 5.3 (*n* = 3)	14.6 ± 12.4 (*n* = 4)	16.8 ± 8.1 (*n* = 4)	13.3 ± 9.3 (*n* = 4)	22.5 ± 8.3 (*n* = 4)	0.652	0.200	0.754
UA (mg/dL)	3.8 ± 0.6 (*n* = 5)	7.1 ± 2.7 (*n* = 6)	4.7 ± 0.6 (*n* = 5)	7.1 ± 2.9 (*n* = 3)	4.7 ± 2.1 (*n* = 4)	7.6 ± 2.6 (*n* = 4)	4.5 ± 1.9 (*n* = 4)	8.9 ± 1.9 (*n* = 4)	0.669	< 0.0001	0.797
IgA (μg/mL)	222 ± 123 (*n* = 5)	193 ± 95 (*n* = 6)	459 ± 113 (*n* = 5)	242 ± 127 (*n* = 3)	354 ± 259 (*n* = 4)	236 ± 79 (*n* = 4)	415 ± 214 (*n* = 4)	196 ± 39 (*n* = 4)	0.183	0.010	0.435

AST: aspartate transaminase; ALT: alanine transaminase; UA: uric acid; IgA: immunoglobulin A. Values are presented as mean ± SD. [1] The calculated *p*-values using DON (D) and sex (S) as the main effects in factorial model.

Table S3. The impact of deoxynivalenol (DON) on relative weights (% of body weight, BW) of liver, heart, and spleen in Taiwan country chickens at 16 weeks of age.

Relative weight (% of BW)	Treatment (DON, mg/kg)								*p*-Values [1]		
	0		2		5		10				
	♀	♂	♀	♂	♀	♂	♀	♂	D	S	D × S
Heart	0.40 ± 0.07 (*n* = 6)	0.53 ± 0.09 (*n* = 6)	0.48 ± 0.11 (*n* = 5)	0.49 ± 0.06 (*n* = 3)	0.35 ± 0.04 (*n* = 4)	0.51 ± 0.03 (*n* = 4)	0.43 ± 0.07 (*n* = 4)	0.53 ± 0.07 (*n* = 4)	0.361	0.000	0.216
Liver	2.32 ± 0.39 (*n* = 6)	1.40 ± 0.26 (*n* = 6)	2.21 ± 0.33 (*n* = 5)	1.34 ± 0.19 (*n* = 3)	2.33 ± 0.49 (*n* = 4)	1.39 ± 0.17 (*n* = 4)	1.97 ± 0.19 (*n* = 4)	1.33 ± 0.24 (*n* = 4)	0.470	< 0.0001	0.761
Spleen	0.09 ± 0.02 (*n* = 6)	0.10 ± 0.02 (*n* = 6)	0.08 ± 0.03 (*n* = 5)	0.16 ± 0.01 (*n* = 3)	0.13 ± 0.07 (*n* = 4)	0.18 ± 0.10 (*n* = 4)	0.09 ± 0.06 (*n* = 4)	0.14 ± 0.01 (*n* = 4)	0.038	0.018	0.616

Values are presented as mean ± SD. [1] The calculated *p*-values using DON (D) and sex (S) as the main effects in factorial model.

Table S4. Detection of cell proliferation marker PCNA, apoptosis signals by TUNEL assay, and DNA damage related marker γ-H2AX in spleen sections of Taiwan country chickens exposed to deoxynivalenol (DON) at 16 weeks of age.

Positive cells (% of total cells)	Treatment (DON, mg/kg)								*p*-Values[1]		
	0		2		5		10				
	♀	♂	♀	♂	♀	♂	♀	♂	D	S	D × S
PCNA	7.5 ± 1.2 [bc] (*n* = 4)	10.5 ± 1.2 [ab] (*n* = 4)	9.1 ± 1.0 [ab] (*n* = 3)	10.7 ± 1.6 [ab] (*n* = 3)	9.5 ± 1.7 [ab] (*n* = 3)	12.0 ± 2.9 [a] (*n* = 3)	4.0 ± 1.4 [c] (*n* = 3)	12.7 ± 1.4 [a] (*n* = 3)	0.080	< 0.0001	0.004
TUNEL	2.5 ± 0.5 (*n* = 4)	4.0 ± 1.6 (*n* = 4)	3.3 ± 0.7 (*n* = 3)	5.8 ± 1.8 (*n* = 3)	5.5 ± 1.1 (*n* = 3)	5.4 ± 2.9 (*n* = 3)	5.0 ± 1.8 (*n* = 3)	5.9 ± 0.8 (*n* = 3)	0.043	0.060	0.544
γ-H2AX	6.6 ± 1.9 (*n* = 4)	8.3 ± 1.8 (*n* = 4)	10.1 ± 2.6 (*n* = 3)	6.4 ± 1.6 (*n* = 3)	8.4 ± 1.9 (*n* = 3)	8.4 ± 0.3 (*n* = 3)	11.8 ± 2.6 (*n* = 3)	9.8 ± 2.8 (*n* = 3)	0.048	0.351	0.120

PCNA: proliferating cell nuclear antigen; TUNEL: terminal deoxynucleotidyl transferase dUTP nick end labeling. Values are presented as mean ± SD. [1] The calculated *p*-values using DON (D) and sex (S) as the main effects in factorial model. [a,b,c] Means within a row without a common superscript differ significantly ($p < 0.05$).

Table S5. The impact of deoxynivalenol (DON) on villus morphology of the small intestine in Taiwan country chickens at 16 weeks of age.

Villus height (μm)	Treatment (DON, mg/kg)								*p*-Values[1]		
	0		2		5		10				
	♀	♂	♀	♂	♀	♂	♀	♂	D	S	D × S
Jejunum	1101 ± 225 (*n* = 6)	1041 ± 114 (*n* = 6)	1064 ± 155 (*n* = 5)	1212 ± 82 (*n* = 3)	1022 ± 321 (*n* = 4)	1021 ± 207 (*n* = 4)	1083 ± 228 (*n* = 4)	1064 ± 183 (*n* = 4)	0.808	0.920	0.730
Ileum	797 ± 223 (*n* = 6)	654 ± 195 (*n* = 6)	619 ± 179 (*n* = 5)	621 ± 48 (*n* = 3)	848 ± 140 (*n* = 4)	398 ± 108 (*n* = 4)	769 ± 187 (*n* = 4)	552 ± 70 (*n* = 4)	0.351	0.002	0.079

Values are presented as mean ± SD. [1] The calculated *p*-values using DON (D) and sex (S) as the main effects in factorial model.
